# Extraction and Evaluation of Outer Membrane Vesicles from Two Important Gut Microbiota Members, *Bacteroides fragilis* and *Bacteroides thetaiotaomicron*

**DOI:** 10.22074/cellj.2020.6499

**Published:** 2019-12-15

**Authors:** Sara Ahmadi Badi, Arfa Moshiri, Fatemeh Ettehad Marvasti, Mojtaba Mojtahedzadeh, Vida Kazemi, Seyed Davar Siadat

**Affiliations:** 1. Department of Biology, Science and Research Branch, Islamic Azad University, Tehran, Iran; 2. Cancer Department, Gastroenterology and Liver Diseases Research Center, Research Institute for Gastroenterology and Liver Diseases, Shahid Beheshti University of Medical Sciences, Tehran, Iran; 3. Laboratory of Experimental Therapy in Oncology, G. Gaslini Children’s Hospital, Genoa, Italy; 4. Microbiology Research Centre, Pasteur Institute of Iran, Tehran, Iran; 5. Department of Pharmacotherapy, Faculty of Pharmacy, Tehran University of Medical Sciences, Tehran, Iran; 6. Medicinal Plants Research Centre, Faculty of Pharmacy, Tehran University of Medical Sciences, Tehran, Iran; 7. Mycobacteriology and Pulmonary Research Department, Pasteur Institute of Iran, Tehran, Iran

**Keywords:** *Bacteroides fragilis*, *Bacteroides thetaiotaomicron*, Gut Microbiota

## Abstract

**Objective:**

The gastrointestinal tract (GI) is colonized by a complex microbial community of gut microbiota. Bacteroides spp. have significant roles in gut microbiota and they host interactions by various mechanisms, including outer membrane vesicle (OMVs) production. In the present study, we extracted and assessed *Bacteroides fragilis (B. fragilis)* and *Bacteroides thetaiotaomicron (B. thetaiotaomicron)* OMVs in order to evaluate their possible utility for *in vivo* studies.

**Materials and Methods:**

In this experimental study, OMVs extraction was performed using multiple centrifugations and tris-ethylenediaminetetraacetic acid (EDTA)-sodium deoxycholate buffers. Morphology, diameter, protein content, profile, and lipopolysaccharide (LPS) concentrations of the OMVs were assessed by scanning electron microscopy (SEM), nanodrop, Bradford assay, sodium dodecyl sulphate-polyacrylamide gel electrophoresis (SDS-PAGE), and the Limulus Amoebocyte Lysate (LAL) test, respectively. Zeta potential (ζ-P) was also assessed. The viability effect of OMVs was assessed by the 3-(4,5-dimethylthiazol-2-yl)-2, 5-diphenyltetrazolium bromide (MTT) assay in Caco-2 cells.

**Results:**

Spherical OMVs with diameters of 30-110 nm were produced. The OMVs had different protein profiles. The LPS concentrations of the *B. fragilis* and *B. thetaiotaomicron* OMVs were 1.80 and 1.68 EU/mL, respectively. ζ-P of the *B. fragilis* OMVs was -34.2 mV and, for *B. thetaiotaomicron*. it was -44.7 mV. The viability of Caco-2 cells treated with OMVs was more than 95%.

**Conclusion:**

The endotoxin concentrations of the spherical OMVs from *B. fragilis* and *B. thetaiotaomicron* were within the safe limits. Both OMVs had suitable stability in sucrose solution and did not have any cytotoxic effects on human intestinal cells. Based on our results and previous studies, further molecular evaluations can be undertaken to design OMVs as possible agents that promote health properties.

## Introduction

Gut microbiota are a diverse and complicated microbial community that colonize the gastrointestinal tract (GI) (1). The gut microbiota have beneficial roles in the host that include colonization resistance, assist with digestion, harvest energy from the diet, metabolism of nutrients, and immune system regulation (2, 3). This microbial community consists of bacteria, archea, viruses, fungi, and protozoa (4). Bacteria are the dominant microbial population. Bacteroidetes and Firmicutes constitute two major bacterial phyla in gut microbiota (5). Bacteroidetes are gram-negative bacteria are abundant and diverse in gut microbiota (6). These bacteria are found at high frequencies (up to 10^11^ cells/g) in intestinal material (7). Bacteroidetes are important in host metabolism since they degrade proteins and complex carbohydrates (8). Moreover, the *Bacteroides spp.* affect function of the immune system, specifically, the tolerance for intestinal commensal bacteria (9). *Bacteroides fragilis (B. fragilis)* and *Bacteroides thetaiotaomicron (B. thetaietomicron)* affect gut microbiota-host interactions as they contain broad metabolic and immune regulating potentials (10). Various strategies are employed by *Bacteroides spp.* in their interaction with the host, including production of metabolites (such as short chain fatty acids) and outer membrane vesicles (OMVs) (11, 12).

OMVs are nano-sized vesicles (20 to 250 nm) secreted by gram-negative bacteria under various conditions during all growth phases (13). The bacterial OMVs were first reported in the 1970s when *Escherichia coli (E. coli)* OMVs were identified in E. coli cultures grown under lysine-limiting conditions (14-17). Thereafter, it has been found that bacterial vesiculation occurs in planktonic cultures, biofilms, and *in vivo* (14). These spherical particles originate from the bacterial outer membrane and contain a wide range of compounds, such as lipopolysaccharide (LPS), outer membrane proteins (OMPs), phospholipids, periplasmic components, DNA, RNA, hydrolytic enzymes, and signaling molecules (18, 19). OMVs play a role in bacterial interactions with the environment. These particles are considered interesting bacterial components due to their participation in numerous processes, including pathogenesis, bacterial survival under stress, and regulation of prokaryote-prokaryote and prokaryote-eukaryote communications (11, 18- 20).

In the normal state, the gut microbiota-host interactions are balanced due to desirable functions of the gut barrier. Many factors such as intestinal epithelial cell integrity, tight junction proteins, and the mucus layer maintain proper gut barrier functions that control gut microbiota-host interactions (8, 21). In recent studies, it was demonstrated that beneficial intestinal commensal bacteria might have adverse effects on the host, while their OMVs maintain beneficial effects on the host functions in leaky gut syndrome, which is characterized by disruption of gut barrier integrity and increased intestinal epithelial cell permeability (21, 22).

The roles of OMVs in gut microbiota homeostasis and host functions are under investigation. In this regard, the study of OMVs production from key gut microbiota members and their properties could contribute to an understanding of the gut microbiota-host interactions. Accordingly, in the present study, the OMVs from two important gut microbiota members,* B. fragilis* and *B. thetaiotaomicron*, were extracted and their physicochemical properties (size, morphology, protein concentration/bands, LPS concentration, and surface charge) were evaluated. Finally, the OMVs effect on the viability of the Caco-2 cell line, as a human gastrointestinal epithelial cell model, was assessed.

## Materials and Methods

### Bacterial strains and growth conditions

In this experimental study, *B. fragilis* ATCC 23745 and *B. thetaiotaomicron* CCUG 10774 were grown either on blood agar plates that contained 5% defibrinated horse blood or brain heart infusion (BHI) broth supplemented with hemin (5 µg/ml) and menadione (1 µg/ml), and incubated at 37˚C under anaerobic conditions (80% N_2_, 10% Co_2_, and 10% H_2_) using an Anoxomat™ MARK II system (10).

### Outer membrane vesicle purification

After an overnight incubation under anaerobic conditions, OMVs were isolated as described previously (23). Briefly, 500 mL of the bacterial cultures were centrifuged at 6000 g at 4˚C. The cell pellets were washed twice in phosphatebuffered solution (PBS). Then, the cell pellets were resuspended in a 9% sodium chloride solution. The cell suspensions were homogenized and concentrated by centrifugation at 2900 g for 1 hour at 4˚C. The total wet weight of cell pellets was calculated and resuspended in 7.5 times the wet weight of 0.1 M tris-10 mM ethylenediaminetetraacetic acid (EDTA) buffer (Sigma-Aldrich, USA). The vesicles were extracted by the addition of 1/20^th^ the volume of 0.1 M Tris, 10 mM EDTA, and sodium deoxycholate (100 g/L) buffer (Merck, Germany). OMVs were separated from cell debris at 20 000 g for 60 minutes at 4˚C. The supernatant that contained the vesicles was centrifuged at 20 000 g for 120 minutes at 4˚C in order to concentrate the vesicles. The pellet was resuspended in 10 mM EDTA, 0.1 M Tris, and sodium deoxycholate (5 g/L) buffer, and the suspension was centrifuged again at 20000 g for 120 minutes at 4˚C. The concentrated OMVs were resuspended in a 3% sucrose solution. Finally, the suspension was filtered through a 0.22-µm polyvinylidene difluoride filter (Millipore, Billerica, MA, USA).

### Scanning electron microscopy

The OMVs were fixed with 2.5% glutaraldehyde and 2% paraformaldehyde in PBS (Sigma-Aldrich, USA). After washing with PBS, dried samples were coated with gold by a sputter coater (SBC-12, KYKY, China) using a physical vapor deposition method. The prepared samples were examined by SEM (KYKYEM3200, KYKY, China) (24).

### Determination of the outer membrane vesicle protein content and pattern

To estimate the amount of total proteins, purified OMVs were analyzed using a NanoDrop spectrophotometer (Thermo Scientific, Wilmington, DE, USA) and the Bradford assay with coomassie brilliant blue, at 590 nm. The protein contents of B. *fragilis* and *B. thetaiotaomicron* OMVs were separated by SDS-PAGE on 12% gels that were stained with coomassie brilliant blue (24).

### Quantification of outer membrane vesicle endotoxins

The content and biological activity of the OMVs endotoxins was measured using the Pierce™ LAL Chromogenic Endotoxin Quantitation Kit (Thermo Scientific, USA) according to the manufacturer’s instructions. Briefly, the microplate was incubated for 10 minutes at 37˚C. We dispensed 50 μL each of the samples and standards into the microplate wells and allowed them to incubate for 5 minutes at 37˚C. Then, 50 μL of LAL was added to each well. The plate was incubated at 37˚C for 10 minutes. We added 100 μL of substrate solution to the microplate and incubated it for 6 minutes at 37˚C. Finally, 50 μL of stop reagent (25% acetic acid) was added to each well and we measured the optical density (OD) the wells at 405-410 nm on a plate reader. The amount of endotoxin in the samples was calculated using the standard curve (25).

### Zeta (ζ-P) potential measurement

The OMVs were prepared by sonication in 35 kHz for 3 minutes (Bandelin ultrasonic bath). The zeta (ζ-P) potential of the OMVs was assessed using a Malvern Zetasizer Nano ZEN3600 (Malvern Instruments, United Kingdom).

### 3-(4,5-dimethylthiazol-2-yl)-2, 5-diphenyltetrazolium bromide assay

The human epithelial cell line, IBRC C10094 Caco- 2 (Iranian Biological Resource Centre) was cultured in Dulbecco’s modified eagle medium (DMEM/high glucose, Gibco, USA), supplemented with 10% fetal bovine serum (FBS, Gibco USA), 1% non-essential amino acids (Gibco, USA), and 1% penicillin/ streptomycin (Gibco USA) and incubated at 37˚C in a 5% CO_2_ atmosphere. Caco-2 cells were seeded at a density of 2×10^4^ cells/well in a 96-well culture plate and incubated overnight before the OMVs treatment. The cells were treated with OMVs (50 µg/ml) and incubated for 24 hours. The cell culture medium was discarded and replaced by fresh medium. After 4 hours of incubation, the cells were incubated with 100 µl medium with MTT for 4 hours. After incubation, the medium was removed and 100 µl dimethyl sulfoxide (DMSO) was added to each well to dissolve the formazan crystals that formed in the living cells. The absorbance was measured at 570 nm (26).

## Results

**Fig 1:**
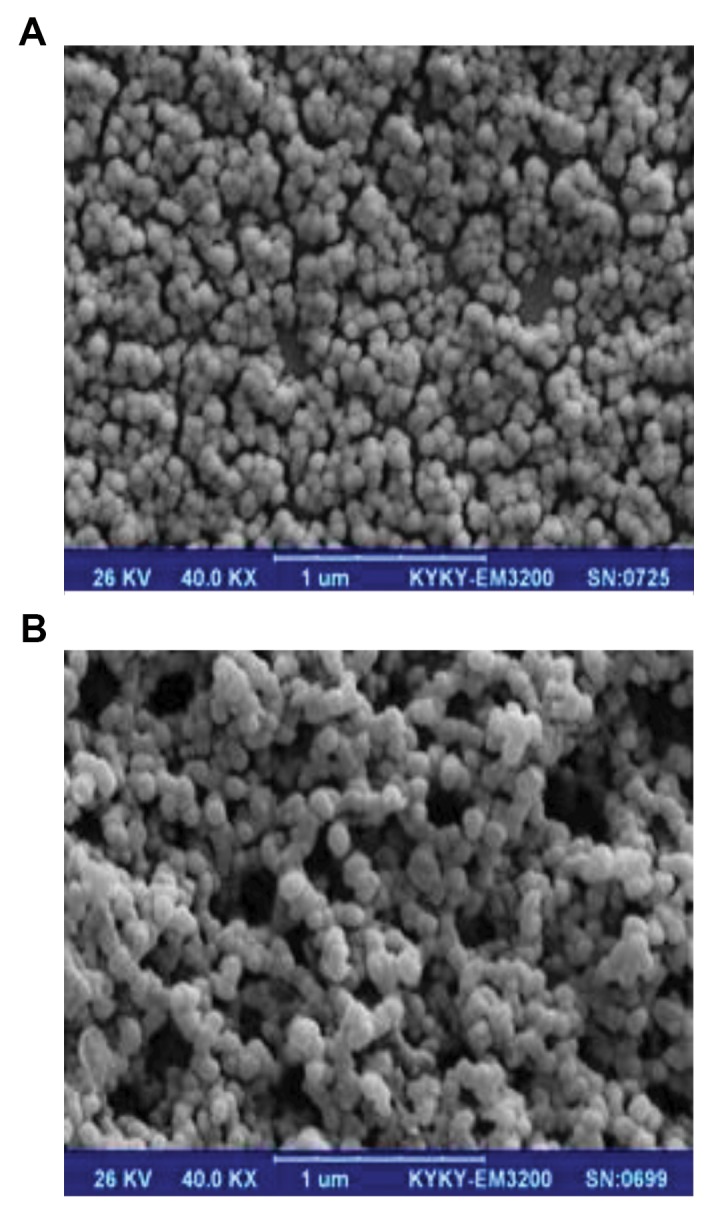
Scanning electron microscopy (SEM) of outer membrane vesicles (OMVs). **A.** *Bacteroides fragilis (B. fragilis)* and ***B.** Bacteroides thetaiotaomicron (B. thetaiotaomicron)* derived OMVs (SEM at magnification: ×40 KX).

*B. fragilis* and *B. thetaiotaomicron* produced spherical OMVs with diameters of 30-110 nm, as determined by SEM (Fig .1).

**Fig 2:**
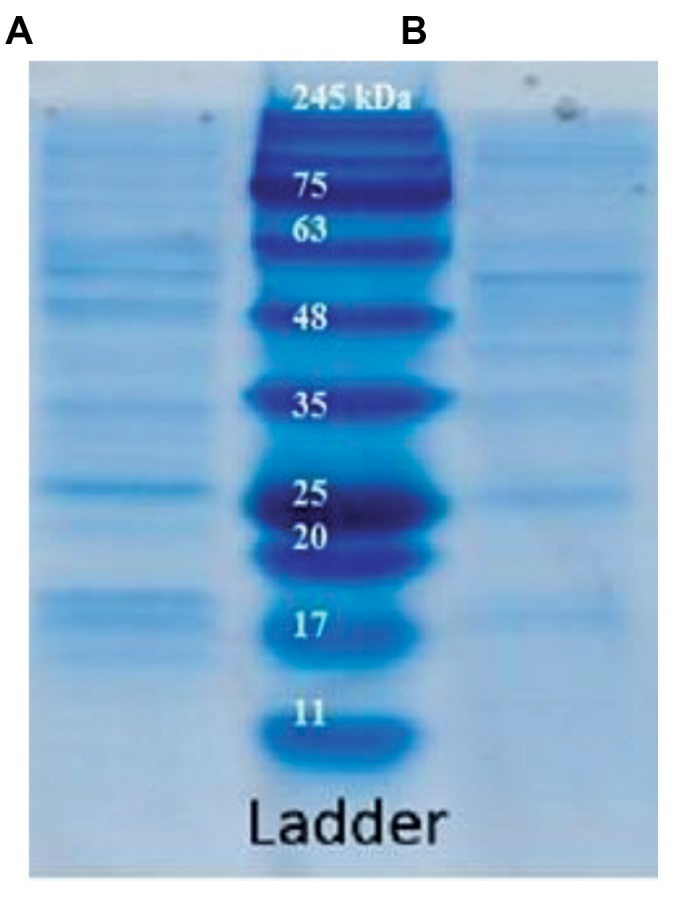
Sodium dodecyl sulfate polyacrylamide gel (SDS-PAGE) showing the protein profile of outer membrane vesicles. The protein bands of **A.** *Bacteroides fragilis (B. fragilis)* and **B.** *Bacteroides thetaiotaomicron (B. thetaiotaomicron)* derived- outer membrane vesicles (OMVs) at 0.35 mg/ ml and 0.45 mg/ml protein concentrations, respectively.

Our results based on SDS-PAGE showed that OMVs derived from *B. fragilis* and* B. thetaiotaomicron* had different protein profiles. After OMV purification, we measured the protein content of these particles by using a NanoDrop and the Bradford assay. The protein content of the OMVs from *B. fragilis *was 0.35 mg/ml and it was 0.45 mg/ml for *B. thetaiotaomicron*. SDS-PAGE analysis showed different protein bands of OMVs, especially between 11-17 KDa, in *B. fragilis* and *B. thetaiotaomicron* (Fig .2).

**Fig 3:**
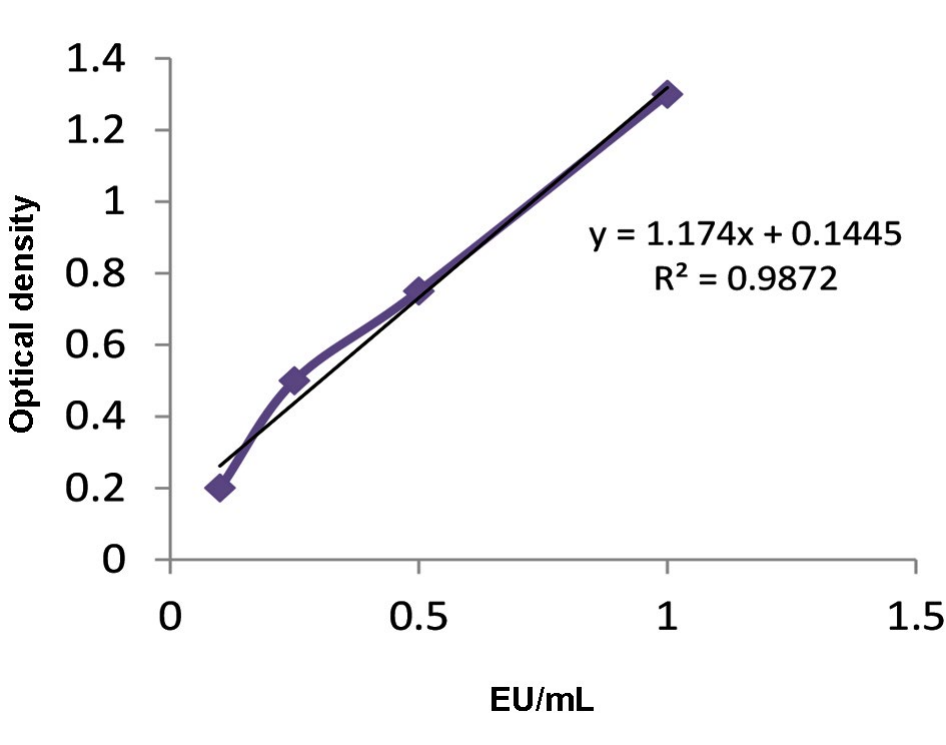
The standard curve of the limulus amoebocyte lysate (LAL) test to determine the endotoxin levels of *Bacteroides fragilis (B. fragilis) *and *Bacteroides thetaiotaomicron (B. thetaiotaomicron) *derived-outer membrane vesicles (OMVs).

The LAL test was performed to detect and quantify the amount of endotoxin from the OMVs. By using the standard curve (Fig .3), we determined that the LPS concentration of OMVs from *B. fragilis* was 1.80 EU/ mL and it was 1.68 EU/mL for *B. thetaiotaomicron* derived-OMVs.

ζ-P of these vesicles was measured by electrophoretic light scattering (ELS). Both *B. fragilis* and *B. thetaiotaomicron* OMVs had negative surface charges of -34.2 and -44.7 mV, respectively (Figes.4, 5).

**Fig 4:**
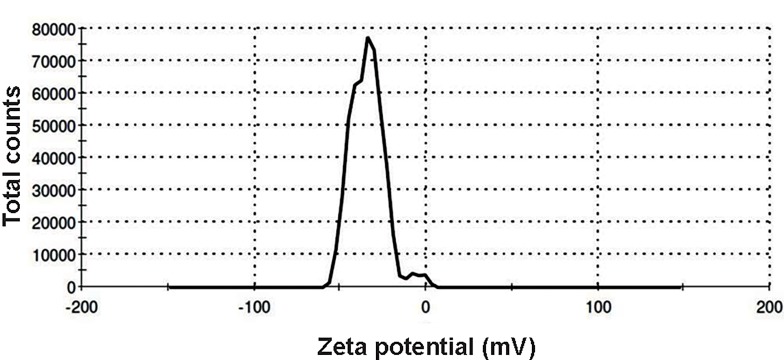
The zeta potential (ζ-P) distribution of *Bacteroides fragilis (B. fragilis)* derived-outer membrane vesicles (OMVs).

**Fig 5:**
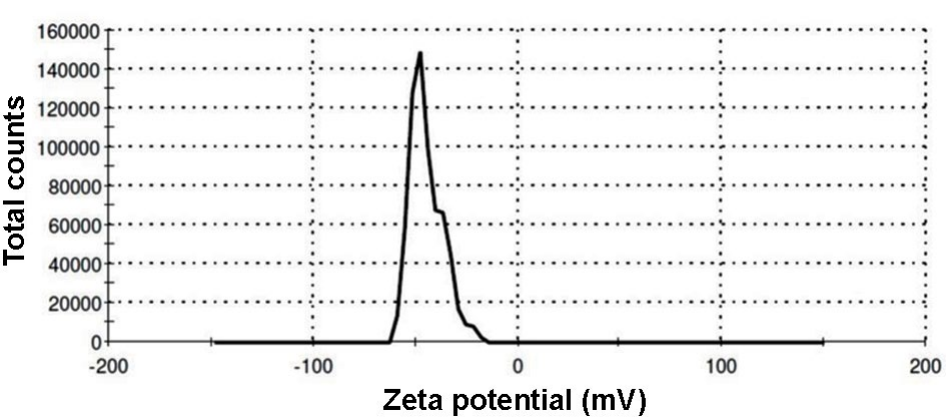
The zeta potential (ζ-P) distribution of *Bacteroides thetaiotaomicron (B. thetaiotaomicron) *derived-outer membrane vesicles (OMVs).

The human intestinal epithelial cell line, Caco-2, was used to study the effects of *B. fragilis* and* B. thetaiotaomicron* derived OMVs on epithelial cell viability. MTT assays showed more than 95% viability of Caco-2 cells treated with both OMVs at a specific concentration.

## Discussion

It is well documented that gut microbiota has a profound effect on host health and diseases. The communication between gut microbiota and the host is mainly dependent on the microbial released factors, which could access intestinal epithelial cells (27). OMVs have considerable roles in putative communication since they interact with host cells through their various components, such as bacterial outer membrane determinants, hydrolytic enzymes, and signaling molecules (11). Among numerous microbial species that colonize the GI, the Bacteroidetes comprise the most gram-negative bacteria. Both *B. fragilis* and *B. thetaiotaomicron* have important roles in gut microbiota which produce OMVs that are delivered to distant targets of the host (6). In the present study, we aimed to extract and evaluate the characteristics of OMVs from *B. fragilis* and *B. thetaiotaomicron*.

OMVs originate from the outer membrane of gramnegative bacteria and are released to the extracellular milieu as small particles by bilayer spherical shaped vesicles. Several pathogenic and non-pathogenic bacteria are proposed to produce OMVs, such as *Mycobacterium tuberculosis* (28), Neisseria meningitides (29), and E.coli (30). B. fragilis and B. thetaiotaomicron are two predominant gut microbiota which produces OMVs to exert beneficial effects on the host, including mediation of anti-inflammatory responses, immune tolerance to the gut microbiota, and homeostasis (31). Stentz et al. (32) and Elhenawy et al. (10) purified *B. fragilis* and *B. thetaiotaomicron* OMVs by ultrafiltration and ultracentrifugation, respectively. In the present study, *B. fragilis* and *B. thetaiotaomicron* derived-OMVs were extracted by sequential centrifugation and buffers that contained sodium deoxycholate based on the modified method of Claassen et al. (23).

The evaluation of the physicochemical properties of OMVs is a characteristic examination marker. Morphology, size, and protein content of *B. fragilis* and *B. thetaiotaomicron* OMVs were reported by transmission electron microscopy (TEM) and SDS-PAGE, respectively (10, 32). According to the current study, spherical shaped vesicles from *B. fragilis* and *B. thetaiotaomicron* were observed that ranged in diameter from 30 to 110 nm with SEM. These organisms produced OMVs with different protein bands by SDS-PAGE.

Recently, several studies have reported the potential applications of OMVs as novel vaccine adjuvants and cancer immunotherapeutic agents (33, 34). As mentioned, a previous work and our studies (unpublished data) have noted potent roles for *B. fragilis* and *B. thetaiotaomicron* derived-OMVs, which has made them promising agents to improve targeted pathways of hosts, including the immune and metabolic systems. For this propose, we performed this study to evaluate endotoxin level, surface charge, and cytotoxicity effect on human intestinal epithelial cells.

Bacteroidetes phylum is the major LPS-producing bacteria in gut microbiota. In this study, the LPS concentration of OMVs was assessed by the LAL test. This test, which is known as the bacterial endotoxin test (BET), is performed for over 90% of pyrogenic tests. This measurement is significant for OMVs application as therapeutic agents. To our knowledge, this is the first report of the LPS concentration of *B. fragilis* and *B. thetaiotaomicron* OMVs, which was identified as 1.80 and 1.68 EU/mL, respectively. These obtained values are less than the defined tolerable endotoxin amount according to the United States Pharmacopeia (35).

Surface charges of vesicles are measured and reported as ζ-P potentials. The particle charge has a determinative role in the physical stability of suspensions. Generally, particles which have ζ-P potentials more positive than +30 mV or more negative than -30 mV are stable (36). Measurement of ζ-P potential could provide information about the aggregation and stability of OMVs in sucrose solution. In this study, the ζ-P potentials of *B. fragilis* and *B. thetaiotaomicron* derived OMVs were measured for the first time. These values were found to be less aggregated and thus more stable in sucrose solution.

Intestinal epithelial cells are the interface between gut microbiota and host interactions. Therefore, the effects of *B. fragilis* and *B. thetaiotaomicron* derived OMVs on Caco-2 cell viability (a human intestinal epithelial cell model) were assessed. Our result showed that these particles did not have cytotoxic effects on Caco-2 cells at a specific concentration.

## Conclusion

According to our results, *B. fragilis* and *B. thetaiotaomicron* spherical nanosized OMVs have different protein profiles, a safe endotoxin content, and no cytotoxic effect at a specific concentration on a human epithelial cell line. They could be new promising agents to suggest their utility in *in vivo* studies as the novel therapeutic candidates. However, further molecular investigations are needed to explore their roles in more details.
